# Ionizing radiation-induced DNA injury and damage detection in patients with breast cancer

**DOI:** 10.1590/S1415-475738420150019

**Published:** 2015

**Authors:** Gissela Borrego-Soto, Rocío Ortiz-López, Augusto Rojas-Martínez

**Affiliations:** 1 Departamento de Bioquímica y Medicina Molecular, Facultad de Medicina, Universidad Autónoma de Nuevo León, Monterrey, Nuevo León, Mexico; 2 Centro de Investigación y Desarrollo en Ciencias de la Salud, Universidad Autónoma de Nuevo León, Monterrey, Nuevo León, Mexico

**Keywords:** breast cancer, ionizing radiation, DNA damage, DNA double strand break, DNA repair analysis

## Abstract

Breast cancer is the most common malignancy in women. Radiotherapy is frequently used in patients with breast cancer, but some patients may be more susceptible to ionizing radiation, and increased exposure to radiation sources may be associated to radiation adverse events. This susceptibility may be related to deficiencies in DNA repair mechanisms that are activated after cell-radiation, which causes DNA damage, particularly DNA double strand breaks. Some of these genetic susceptibilities in DNA-repair mechanisms are implicated in the etiology of hereditary breast/ovarian cancer (pathologic mutations in the *BRCA* 1 and 2 genes), but other less penetrant variants in genes involved in sporadic breast cancer have been described. These same genetic susceptibilities may be involved in negative radiotherapeutic outcomes. For these reasons, it is necessary to implement methods for detecting patients who are susceptible to radiotherapy-related adverse events. This review discusses mechanisms of DNA damage and repair, genes related to these functions, and the diagnosis methods designed and under research for detection of breast cancer patients with increased radiosensitivity.

## Background

Breast cancer is the leading cause of cancer morbidity and death in women in developed countries and countries with emerging economies (Ripperger *et al.*, 2009; Youlden *et al.*, 2012). According to Globocan, 1.67 million new cases of breast cancer were diagnosed in 2012 and ranks as the fifth cause of death from cancer overall (522,000 deaths). A global increase has been estimated to around 16,500 yearly new cases of this neoplasia by 2020. (Knaul *et al.*, 2009)

Radiation therapy is an efficient treatment for cancer. About 50% of patients with malignant breast tumors receive radiation therapy and most patients seem tolerate it, but some suffer severe adverse effects induced by the therapy. This variability of response may be caused by several factors, like age, life style, inflammatory responses, oxidative stress, genetic predisposition and variants in genes involved in the response to radiation-induced DNA damage (Smirnov *et al.*, 2012; Hornhardt *et al.*, 2014). Therefore, it is important to develop new diagnostic techniques for predicting responses to cancer treatment and for identifying patients susceptible to radiation-related toxicity.

Any disturbance that results in the loss of genomic integrity may induce cell cycle deregulation and uncontrolled cell proliferation. Cells are continuously exposed to DNA damaging agents and have developed mechanisms to respond to genome damage. Double-strand DNA breaks (DSB), although rare, are perhaps the most lethal mechanism and are often produced by ionizing radiation (Pastink *et al.*, 2001; Siever *et al.*, 2003). The BRCA-1 and BRCA-2 proteins are involved in DSB damage repair, and several mutations in these genes increase the risk for developing breast and other neoplasias (Roy *et al.*, 2012).

## Ionizing Radiation-Associated DNA Damage, Radiotherapy and Mechanisms of DNA Repair

### Ionizing radiation effects in the cell

Ionizing radiation is a type of high-energy radiation that is able to release electrons from atoms and molecules generating ions which can break covalent bonds. Ionizing radiation directly affects DNA structure by inducing DNA breaks, particularly, DSBs. Secondary effects are the generation of reactive oxygen species (ROS) that oxidize proteins and lipids, and also induce several damages to DNA, like generation of abasic sites and single strand breaks (SSB). Collectively, all these changes induce cell death and mitotic failure.

Ionizing radiation can be divided into X-rays, gamma rays, alpha and beta particles and neutrons. Quiescent and slowly dividing cells are less radiosensitive, like those constituting the nervous system, while cells with high proliferation rates are more radiosensitive, like bone marrow, skin, and epithelial cells of the gastro-intestinal tract, among others. The radiation dose is measured in units gray (Gy), a measure of the amount of radiation absorbed by 1 kg of tissue (Dunne-Daly, 1999).

### Radiotherapy

Radiotherapy is a treatment aimed at shrinking the tumor mass or at eliminating residual tumor cells by exposing the tumor to ionizing radiation. Radiotherapy regimes mostly use X- and gamma radiation (Masuda and Kamiya, 2012). Radiation affects tumor and healthy irradiated cells indistinctly. Radiotherapy is used as the standard treatment for breast cancer after mastectomy; but this therapy may be also used prophylactically or palliatively to reduce the risk of tumor recurrence or to relieve symptoms caused by tumor growth and associated metastases, respectively (Delaney *et al.*, 2005). Radiation therapy can be delivered by external-beam radiation or internal radiation. External-beam radiation therapy is created electronically by a linear accelerator which produces photon beams known as X-rays, with electric potentials in the range of 4 to 20 mega Volts. Patients receive radiation doses in daily sessions for several weeks, and the radiation dose may be administered in three different schemes: accelerated fractionation, hyperfractionation and hypofractionation. Accelerated fractionation means a radiation scheme in which the total dose of radiation is divided into small doses, and the treatments are given more than once per day. The total dose of radiation is administered in a shorter period of time (fewer days) compared to standard radiation therapy (weeks). A reduction in the treatment time may reduce the repopulation of tumor cells, resulting in a better locoregional control. In hyperfractioned treatment, the total radiation dose is divided into smaller doses, and it is administered more than once a day; but in the same period as standard radiotherapy (days or weeks). Dose reduction may reduce the toxicity risk, although the total dose is increased. Hypofractionated radiation treatment is given once a day or less often. The total dose is divided into larger doses and is administered over a shorter period than standard radiotherapy. This scheme reduces patient visits and cost, and fewer side effects are noticed when compared to conventional radiation therapy.

The internal radiation therapy, also called brachytherapy, is released from gamma-radiation sources such as radioactive isotopes like ^60^Co and ^137^Cs, which are placed within the patient's body. This type of radiation can deliver high doses of focalized radiation with an electric potential in the range of 0.6 to 1 megaVolt and causes less damage to normal tissues (Patel and Arthur, 2006).

### DNA repair after ionizing radiation

Ionizing radiation causes DSBs directly, but in addition base damages due to indirect effects are also induced. This radiation causes formation of ROS (reactive oxygen species) which are indirectly involved in DNA damage. These ROS generates apurinic / apyrimidinic (abasic) sites in the DNA, SSBs, sugar moiety modifications, and deaminated adducted bases (Redon *et al.*, 2010; Aparicio *et al.*, 2014). When DNA is damaged, the repair machinery of the cell is activated and stops the cell cycle at specific control checkpoints to repair DNA damage and prevent continuation of the cycle. It is known that the intrinsic radiosensitivity of tumor cells is strongly influenced by the cells DSB repair capability (Mladenov *et al.*, 2013). If tumor cells are able to efficiently repair the radiation damage, resistance to radiation develops, enabling cells to survive and replicate. If the damage remains unrepaired, these mechanisms induce programmed cell death or apoptosis to prevent accumulation of mutations in daughter cells (Deckbar *et al.*, 2011; Guo *et al.*, 2011).

As mentioned, ionizing radiation inevitably reaches normal tissue, inducing bystander effects in tumor-adjacent normal cells that may contribute to chromosomal aberrations and to increase the risk for new malignancies. High doses of radiation may produce toxicity and reduce the patient's prognosis (Brown *et al.*, 2015). Individual radiation treatment based on DSB repair capability could predict toxicity to surrounding tissues, thereby improving treatment safety. DSB repair capability depends not just on gene integrity, but also on gene expression. In addition to germinal mutations affecting genes like *BRCA* 1 and 2 or other related genes, genetic and epigenetic mechanisms may reduce or abrogate the expression of genes involved in DSB repair (Bosviel, *et al.*, 2012). The DNA repair capability could be relevant to decide on the appropriate treatment for cancer patients, and functional tests may provide valuable information for these clinical decisions.

### DSB repair pathways

**Figure 1:**
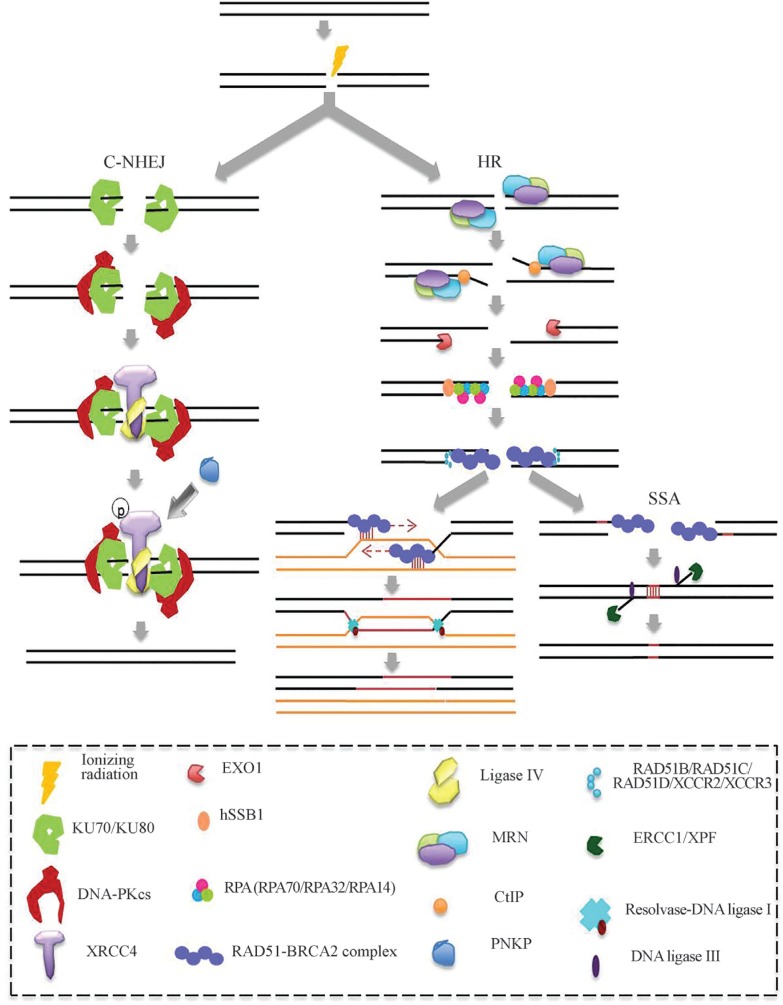
DSB repair pathways. In NHEJ, the KU70/KU80 heterodimer binds to the DSB, protects it from degradation by exonucleases, and acts as a repressor of HR. The KU70/80 heterodimer recruits and activates the DNA-PKcs and KU70 interacts with XRCC4. Then, the DNA ligase IV interacts with the KU heterodimer to ligate the DNA ends. If required for ligation, PNKP binds to phosphorylated XRCC4 to process the DNA ends. In the HR pathway the MRN complex is recruited at the DSB ends and CtIP binds to the MRN complex activating an exonuclease activity which creates single strand segments at the borders of the DSB that are extended by the EXO1 3′- 5′ exonuclease. Then, hSSB1 binds to free ends and RPA (an heterometic complex formed by RPA70, RPA32 and RPA14) protects against degradation. RPA is replaced by RAD51-BRCA2. RAD51 nucleoprotein searches for and invades the homologues sequences, from sister chromatid, to form a Holliday junction. The sister chromatids are joined by cohesin proteins to facilitate the interconnection of the DSB to the homologous recombination. Subsequently, RAD51 is removed leaving a free 3′-OH and DNA is synthesized by the DNA polymerase δ using the homologous chromatid as a template. Resolvase enzymes solve the Holliday junction and the DNA ends are joined by DNA ligase I. The SSA pathway is not conservative and depends on the presence of repeated sequences flanking the DSB. In this mechanism, the MRN complex joined to CtIP cleaves the 5′-end of one strand of DNA to expose microhomology sequences. Homologous sequences are aligned, while nonaligned regions are removed by the ERCC1/XPF nucleases. Then, DNA ends are joined by DNA ligase III.

DSB repair is achieved in three ways: non-homologous end joining (NHEJ), conservative homologous recombination (HR) and single-strand alignment, also called non-conservative homologous recombination (SSA) (Langerak and Russell, 2011). HR is considered an error-free mechanism because it uses an undamaged DNA guide strand to repair the DSB, and the original DNA is reconstituted without loss of genetic information, but this mechanism proceeds slowly and is only exerted at the S/G2 phases of the cell cycle. NHEJ and SSA are considered error-prone and mutagenic mechanisms because the processing of DNA ends may incur in loss or modification of genetic information at the repaired DSB ends. NHEJ is the most common mechanism of DSB repair in eukaryotic cells. In this mechanism, the DNA strands at the DSB are cut or modified, and the ends are ligated together regardless of homology, generating deletions or insertions. Although this process is error-prone, this mechanism can fix the DNA damage quickly, because it is not restricted to a single cell cycle phase, thus preventing increased genetic instability (Do *et al.*, 2014). These mechanisms are detailed below and in the Figure 1. The main proteins involved in the early steps of DSB detection, chromatin remodeling and DNA repair are listed in Table 1.

**Table 1:** DNA repair and cell cycle control genes.

Gene	Name	Function	Cromosomal location
*AKT1*	v-akt murine thymoma viral oncogene homolog 1	Serine/threonine kinase. Regulates components of the apoptotic machinery.	14q32.32
*ATM*	Ataxia telangiectasia mutated	Serine threonine protein kinase. Activates cell cycle checkpoints upon DSB induction acting as a DNA damage sensor.	11q22-q23
*BAP1*	BRCA1 associated protein-1 (ubiquitin carboxy-terminal hydrolase)	Binds to BRCA1. Involved in cell cycle, response to DNA damage and chromatin dynamics.	3p21.1
*BIRP1*	BRCA1 protein interaction with c-terminal helicase	Receptor-interacting protein forming a complex with BRCA1. Active during DSB repair.	17q22.2
*BRCA1*	Breast cancer 1	DNA repair,ubiquitination and transcriptional regulation to maintain genomic stability. Induces cell cycle arrests after ionizing irradiation.	17q21
*BRCA2*	Breast cancer 2	Involved in DSB repair and/or homologous recombination in meiosis.	13q12
*CDKs*	Cell Division Protein Kinase	Cell cycle kinases.	10q21.2
*CDKN1B*	Cyclin-dependent kinase inhibitor 1B	Cell-cycle progression at G1.	12p13.1-p12
*CCND1*	Cyclin D1	Regulates cell cycle during G1/S, also interacts with a network of repair proteins including RAD51 to regulate HR	11q13
*CCND3*	Cyclin D3	Regulates G1/S transition in cell cycle	6p21.1
*RBBP8*	Retinoblastoma Binding Protein	Endonuclease that functions with MRX complex in the first step of the DSB repair.	18q11.2
*EP300*	3 00 kDa E1A-Binding protein gene	Regulates transcription *via* chromatin remodeling. Regulated by acetylation in DNA damage response.	22q13.2
*EXO1*	Exonuclease 1	5’-3’ Exonuclease	1q43
*FGFR2*	Fibroblast growth factor receptor 2	Cell surface tyrosine kinase receptor regulating cell proliferation, migration and apoptosis.	10q25.3-q26
*HIST1H2BC*	Histone cluster 1, H2BC	Core histone playing roles in DNA repair, replication and chromosomal stability.	6p22.1
*H2AX*	H2A Histone Family, Member X	Required for checkpoint-mediated arrest of cell cycle progression in response to low doses of ionizing radiation and for efficient DSB repair when modified by C-terminal phosphorylation.	11q23.3
*KU70*	Thyroid Autoantigen 70 kDa	Binding to DSB ends and inhibition of exonuclease activity at these ends.	22q13.2
*LIG4*	Ligase IV	DNA ligase involved in DNA non-homologous end joining (NHEJ) required for DSB repair.	13q33.3
*LSP1*	Lymphocyte-specific protein 1	Actin binding protein F.	11p15.5
*MDC1*	Mediator of DNA Damage Checkpoint 1	Mediator-adaptor protein in response to DNA damage, active during the S and G2/M phases of cell cycle.	6p21.3
*MLL3*	Myeloid/lymphoid or mixed-lineage leukaemia 3	Part of the ASCOM complex regulated by acetylation to induce expression of p53 targets such as p21 in DNA damage response.	7q36.1
*MRE11*	Meiotic Recombination 11	Endonuclease, exonuclease, MRN/X complex-5.	11q21
*NBN1*	Nibrin	Component of the MRN/X complex. Plays a critical role in the cellular response to DNA damage and the maintenance of chromosome integrity. Regulator of cell cycle checkpoints in meiosis.	8q21.3
*PALB2*	Partner and localizer of BRCA	Critical role in HR repair by recruiting BRCA2 and RAD51.	16p12.1
*PTEN*	Phosphatase and tensin homolog	Tumor suppressor protein. Active in DNA repair through interactions with the Chk1 and the P53 pathways. Regulator of the RAD51 activity.	10q23.3
*RAD50*	RAD50 homolog *Sacharomyces cerevisiae*	Protein involved in DSB repair, required for NHEJ and HR.	5q23-q31
*RAP80*	Ubiquitin Interaction Motif Containing 1	Recognize ubiquitinated H2A and H2AX histones and recruits the BRCA1/BARD1 heterodimer at DSB.	5q35.2
*RB1*	Retinoblastoma	Tumor suppressor protein, mediates cell cycle arrest.	17q22.2
*Rif1*	RAP1 interacting factor homolog (yeast)	Required for cell cycle arrest at S-phase in response to DNA damage.	2q23.3
*RNF168*	RING Finger Protein	E3 ubiquitin-protein ligase required for recruiting repair proteins to DNA damage sites.	3q29
*TGF*β*1*	Transforming growth factor β1	Multifunctional peptides that regulate cell proliferation, migration, adhesion, differentiation, and other functions.	19q13.1
*TopBP1*	Topoisomerase (DNA) II Binding Protein	S-phase checkpoint regulator.	3q22.1
*TOX3*	Tox high mobility group box family member 3	Involved in alteration of chromatin structure.	16q12.1
*TP53*	Tumor protein p53	Tumor suppressor protein, cell cycle arrest, apoptosis, senescence and DNA repair.	17p13
*XLF/Cernunnos*	Non homologous End-Joining Factor	Scaffold protein. Serve as a bridge between XRCC4 and the other NHEJ factors.	2q35
*XRCC4*	X-Ray Repair Complementing Defective	Scaffold protein involved in NHEJ.	5q14.2
*53BP1*	Tumor Protein P53 Binding Protein	Adaptor protein, chromatin reader. Promotes NHEJ.	15q15.3

#### Non-homologous end joining (NHEJ)

Canonical NHEJ (C-NHEJ) is a conservative end-joining process, and this pathway is also essential for V(D)J recombination during T- and B-cell lymphocyte development. NHEJ is not restricted to a particular phase of the cell cycle, but occurs preferentially during the G_0_, G_1_ and the early S phases (Chistiakov *et al.*, 2008; Deckbar *et al.*, 2011; Malu *et al.*, 2012a,b). NHEJ involves ligation of break DNA ends and does not require sequence homology. The first step in the process is the recognition of the DNA ends by the KU heterodimer composed by the KU70 and KU80 proteins. The heterodimer binds to DNA ends and protects them from further degradation (Williams *et al.*, 2014). Crystallographic studies of the KU70/80 heterodimer showed that it adopts a ring-shaped structure encircling the duplex DNA helix which reaches the DNA ends (Walker *et al.*, 2001). The KU subunits are similar in domain organization; they have an amino-terminal von Willebrand domain participating in the KU heterodimerization (Fell and Schild-Poulter, 2012). The KU70/80 heterodimer forms a scaffold at the DNA ends and recruits and activates the DNA-dependent protein kinase catalytic subunit (DNA-PKcs). DNA-PKcs form a pincer-shaped structure which creates a central channel mediating the ability of DNA-PKcs to bind double strand DNA (Sibanda *et al.*, 2010; Davis *et al.*, 2014). Subsequently, the X-ray repair complementing defective repair protein in Chinese hamster cells 4 (XRCC4) interacts with the KU70 subunit and another critical NHEJ scaffolding protein, enabling enzymes to interact with the DSB region. DNA ligase IV directly interacts with the KU heterodimer, an interaction mediated by the tandem BRCA1 C-terminal (BRCT) domains found in the C- terminus of DNA ligase IV (Ochi *et al.*, 2014). Next, the PNKP (polynucleotide kinase-phosphatase) interacts with phosphorylated XRCC4. Structural analysis showed that this scaffold forms filaments interacting with the DNA ends and forms a bridge which stabilizes the ends of the DSB (Hammel *et al.*, 2010; Ochi *et al.*, 2014). It has also been shown that XRCC4 joins to unphosphorylated PNKP, but with less affinity. Other proteins, such as aprataxin, aprataxin and PNKP like factor (APLF), and XRCC4-like factor (XLF) also bind XRCC4.

Usually, DSB ends are irregular and show other defects, like abasic strand segments that must be solved before NHEJ occurs. If phosphate or adenylate groups are present at the DSB ends, DNA end processing may be required for subsequent ligation. PNKP is a kinase/phosphatase responsible for adding phosphate to the 5 ‘OH end and remove the phosphate groups at the 3′ end (Bernstein *et al.*, 2005). Aprataxin is a nucleotide hydrolase and transferase which catalyzes the removal of adenylate groups covalently linked to 5′ phosphate termini (Grundy *et al.*, 2013). When DSB asymmetries must be fixed, the exonuclease Artemis is phosphorylated and binds to DNA-PKcs to trim redundant ends. KU has 5′deoxyribose-5-phosphate (5′-dRP)/AP lyase activity involved in cleaving redundant abasic single strands present at DSB ends (Roberts *et al.*, 2010). The Werner syndrome Rec Q helicase like protein (WRN) joins the KU heterodimer and XRCC4 and stimulate an exonuclease 3′ to 5′ activity (Gu *et al.*, 2010; Malu *et al.*, 2012). Sometimes filling of gaps in the strands at the DSB site is required, and this function may be accomplished by the X family polymerases (μ and λ polymerases) (Capp *et al.*, 2006, 2007).

When DSB ends of two DNA segments are clean and compatible they are ligated by DNA ligase IV (Jahan *et al.*, 2014). Ligase IV activity is stimulated by XRCC4 (Gu *et al.*, 2007). Incompatible ends may be joined by an interaction between ligase IV and XLF.

There is also an alternative NHEJ pathway (A-NHEJ) which is independent of the KU70/KU80 heterodimer activity. In this mechanism, DNA ends are excised by the meiotic recombination 11 protein (MRE11) and the retinoblastoma binding protein 8 (RBBP8, synonymous of CtIP) exonucleases (Gu *et al.*, 2010, Hammel *et al.*, 2010), exposing microhomology regions which can be aligned, allowing the filling of the empty segments by the X family polymerases. Thereafter, XRCC1 and ligase III may complete the end-joining process (Frit *et al.*, 2014). C-NHEJ is a more conservative end-joining process, but its efficacy may be affected by the highly error-prone activity of the A-NHEJ pathway, the adaptability of the C-NHEJ to repair irregular ends, and the incompatibility of some DNA ends (Bétermier *et al.*, 2014).

#### Homologous recombination (HR)

HR for DSB repair requires a homologous DNA sequence provided by the sister homologous chromatid to restore a DSB lesion. Therefore, this process is only active during the S and G2 cell-cycle phases, where this sister chromatid is available as a template (Krejci *et al.*, 2012). HR starts with the binding of the MRN complex to the DSB ends. The MRN complex is constituted by the MRE11 protein, the rad 50 homolog *S. cerevisiae* protein (RAD50) and the nibrin protein (NBS1) (Richard *et al.*, 2011a,b). Then, the 3 ‘ends of the DSB are digested by the exonuclease activity of the MRE11/CtIP to generate free ends at the DSB that are extended by the EXO1 3′- 5′ exonuclease activity (Limbo *et al.*, 2007). Subsequently, the single-strand DNA binding protein 1 (hSSB1) binds to the free 3’ ends and joins the replication protein A (RPA) to protect these free ends from further degradation, to prevent inappropriate annealing that could lead to genomic rearrangements and to prevent hairpin formation (Chen *et al.*, 2013). RPA is a heterotrimeric complex formed by RPA70, RPA32 and RPA14 also involved in the control of DNA replication and repair mechanisms (Sleeth *et al.*, 2007). Then, RPA is replaced by an array of RAD51 proteins assembled to eight BRC domains of the breast cancer 2 (BRCA2) protein and the participation of five additional proteins (RAD51B/RAD51C/RAD51D/XRCC2/XRCC3) (West, 2003). Rad51 is a recombinase which forms a pre-synaptic RAD51-BRCA2 nucleoprotein filament on the DNA (Williams and Michael 2010). The RAD51-BRCA2 nucleoprotein filaments search and invade the homologues sequences to form a Holliday junction structure (Masson *et al.*, 2001). The sister chromatids are joined by the cohesin proteins SMC1, 3, 5 and 6. These proteins facilitate the cohesion of the DSB and the intact homologous strands to propitiate the homologous recombination (Kim *et al.*, 2002, Kong *et al.*, 2014). After the invasion of the sister chromatid (synapses) and the alignment of homologous DNA sequences, RAD51 is removed leaving a free 3′-OH end enabling the repairing DNA synthesis by the DNA polymerase δ in the 3′-5′ direction with the help of resolvases, like the structure-specific endonuclease subunit (MUS81), the essential meiotic structure-specific endonuclease 1 (EME1), and the Holliday junction 5′ flap endonuclease (GEN1) (Constantinou *et al.*, 2002). Once the synthesis of the repaired DNA is completed, these enzymes resolve the Holliday junction and the DNA ends are joined by the DNA ligase I (Matos and West 2014). Although not completely understood, the BRCA1 protein plays an important role in directing the scaffolding of the Rad51-BRCA2 filaments and also interacts with the histone H2AX (described below) during HR repair (O'Donovan and Livingston, 2010).

The HR repair method is considered error-free, because it uses the homologous sequence of the sister chromatid as a template for synthesis. It has been proposed that chromosome condensation makes it difficult to search for homologous sequences in the nucleus, and therefore NHEJ is more frequently employed by cells to repair DSB (Deckbar *et al.*, 2011; Langerak and Russell, 2011). The high fidelity of HR is also proposed to explain the low sensitivity and cellular resistance of cells in S/G2 phase to ionizing radiation. Therefore it is suggested that resistance to radiotherapy is mediates by HR (Somaiah *et al.*, 2013).

#### Single-strand alignment (SSA)

SSA can be regarded as a special form of HR repair. This repair mechanism is not conservative and is dependent on the presence of repeated sequences flanking the DSB. It begins with the cleavage of the 5′-end of one strand of DNA to expose microhomologies. This is mediated by a protein complex composed of the CtIP and the MRN complex, followed by the alignment of the homologous ends. Nonaligned regions are removed by the ERCC1/XPF nucleases (resulting in a loss of nucleotides in the DNA chain) and then, the DNA ends are joined by the DNA ligase III (Salles *et al.*, 2011; Liu *et al.*, 2014). Evidence suggests that SSA repair can elicit the formation of the pathological chromosome translocations related with cancer (Manthey and Bailis, 2010).

### Radiosensitivity in Breast Cancer Patients

Radiosensitivity is the susceptibility of the cells or tissues to ionizing radiation. Some patients may be more sensitive to radiation. Sensitivity results from the toxic effects of radiotherapy resulting in lesions of the patient's normal tissues. These effects may be acute or late, depending on the time of their manifestation. Acute effects occur during the treatment or shortly after and they are usually reversible and occur in rapidly proliferating tissues, like skin, gastrointestinal tract and hematopoietic tissues. Late effects manifest six months or later after the treatment. Late effects can be permanent, mainly affecting slowly proliferating tissues such as kidneys, heart, and the nervous system, and may involve systemic deregulations of the endocrine system (Barnett *et al.*, 2009). Radiation promotes DSB as mentioned above, and this damage is detrimental for genome integrity (Chistiakov *et al.*, 2008; Rübe *et al.*, 2008; Henríquez-Hernández *et al.*, 2011).

Mechanisms of hypersensitivity to ionizing radiation are still unclear, but is estimated that 70% of hypersensitivity cases are due to genetic variants (Turesson *et al.*, 1996). As mentioned above, mutations in the *ATM* gene are associated with extreme hypersensitivity to ionizing radiation (Masuda and Kamiya, 2012), and polymorphisms in genes like *XRCC3* and *RAD51* increase the risk of radiosensitivity (Vral *et al.*, 2011). These genes are also implicated in breast cancer. Mayer *et al.* (2011) analyzed gene expression in peripheral blood lymphocytes of breast and cervical cancer patients. They identified 153 genes altered by ionizing radiation. These genes are involved in cell cycle control and apoptosis in response to radiation. Of these, 67 genes were useful to discriminate between normal reacting patients and subjects with severe radiosensitivity. However, the analyses were performed on lymphocytes, and the authors comment that an analysis of expression in different tissues would be required to define a more precise gene signature (Mayer *et al.*, 2011).

The 7,8-dihydro-8-oxo-2′-deoxyguanosine (8-oxo-dG) base damage is produced by ionizing radiation and is repaired by nucleotide excision followed by removal of this abnormal deoxynucleoside out of the cell (Evans *et al.*, 2010). 8-oxo-dG has been used as a urinary marker of oxidative stress and has been associated with lung cancer (Il'yasova *et al.*, 2012) and gastrointestinal diseases (Ock *et al.*, 2012). It has also been proposed as a marker for radiosensitivity (Erhola *et al.*, 1997, Roszkowski and Olinski, 2012). Haghdoost *et al.* (2001) studied 8-oxo-dG urinary levels in breast cancer patients before and after adjuvant radiotherapy (4 to 6 Gy). Radiosensitive patients showed skin redness in the radiated areas and significantly increased urinary levels of 8-oxo-dG, and these authors proposed the use of this deoxynucleoside as a urinary biomarker for radiosensitivity. This biomarker facilitates the study of individual radiosensitivity, since the abnormal metabolite maybe measured by ELISA (Haghdoost *et al.*, 2001). In a study by Skiöld *et al.* (2013), radiation-induced oxidative stress response was analyzed by the 8-oxo-dG biomarker in serum from *ex-vivo* irradiated leukocytes samples obtained from breast cancer patients that developed severe acute skin reactions (RTOG [Radiotherapy Oncology Group Criteria] grade 3-4) during radiotherapy and from patients with breast cancer showing no early skin reactions after radiotherapy (RTOG grade 0). The authors demonstrated that patients with RTGO grade 0 showed increased extracellular serum levels of 8-oxo-dG, in contrast with the significantly low serum levels observed in patients with RTOG grades 3 and 4, indicating that 8-oxo-dG is a useful biomarker to analyze cellular responses to ionizing radiation (Skiöld *et al.*, 2013). Nonetheless, 8-oxo-dG can also result from cell exposure to oxidative stress by ROS, as may occur when tissues are exposed to environmental pollutants (Hecht, 1999). For these reasons this biomarker is not specific for ionizing radiation but, as in the case of the studies by Skiöld *et al.* (2013), it is helpful as a comparative *ex vivo* test of irradiated cells to define the biological effects of ionizing radiation. Extracellular levels of 8-oxo-dG are appropriate indicators of the cells capability to repair the DNA damage caused by ROS.

Certain phenotypes of breast cancer have been associated with locoregional recurrence (LRR). Brollo *et al.* (2013) suggested that HER2+ tumors are more susceptible to ionizing radiation, while Voduc *et al.* (2010) observed that LRR seemed higher in patients with triple negative marker breast cancer, although the number of LRR events was small. At present, there are no molecular methods to discriminate between patients with high and low LRR (Britten *et al.*, 2013). In addition, there is not enough information regarding the possible adverse effects of radiotherapy that may induce genomic and epigenetic modifications and changes in gene-expression profiles in breast cancer.

Henríquez-Hernández *et al.* (2011) analyzed isolated peripheral blood lymphocytes (PBLs) from patients with advanced breast cancer treated *ex vivo* with high radiotherapy doses to study ionizing radiation resistance. They showed that lymphocytes from patients with low DNA damage and high apoptosis rates had low risks of radiation adverse events.

Studies analyzing the type of repair that occurs when cells are exposed to radiation and the correlation with abnormal expression of certain genes involved in DSB repair have also been conducted. *In vitro* studies of Bca11 (familial breast cancer cell line) and Bca10 (sporadic breast cancer cell line) cell lines showed high NHEJ repair activity and direct HR non-conservative repair in the Bca11 cell line. The Bca10 cell line also showed an increase in non-conservative repair of direct HR, but to a lesser degree than Bca11. Consequently, repair mechanisms in these cell lines may cause deletions in the DNA sequence and cell cycle deregulation (Keimling *et al.*, 2008). These authors performed a study in PBLs from patients with sporadic breast cancer, healthy women with familial risk of breast cancer, and healthy controls, and they demonstrated increased NHEJ and SSA in both, cancer patients and subjects at hereditary risk, *vs.* the healthy controls. This study suggested that these two groups are prone to extended non-conservative DSB repairing mechanisms. Based on these results, Keimling *et al.* (2012) implemented a test to analyze DSB repair *in vitro*.

### Techniques for DSB Repair Analysis

Some tests have been devised to assess DNA damage in response to diverse substances, microorganisms, or environmental conditions. Some of these tests are described below.

### Comet assay

**Figure 2:**
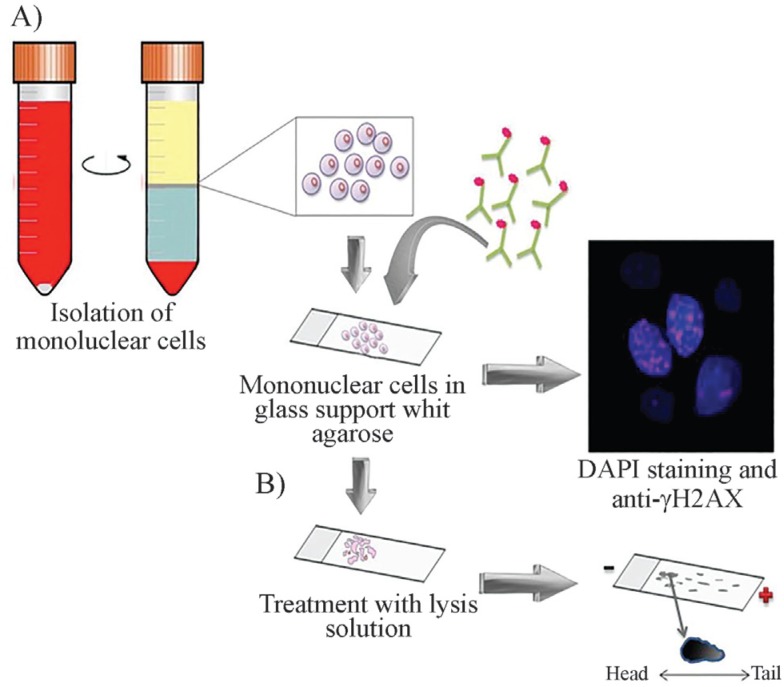
General assays for detecting DNA damage **(A)** Immunohistochemistry with antibodies directed against γ-H2AX: peripheral blood mononuclear cells are isolated, nuclei are stained with DAPI and with antibodies directed at γ-stained H2AX and visualized under fluorescent microscopy. **(B)** Comet assay: the comet assay is also performed on mononuclear cells. The cells are embedded in agarose on a thin glass slide, cells are lysed and incubated in an alkaline solution. Subsequently, DNA fragments are separated by electrophoresis and stained with ethidium bromide. The comet-like image is viewed under a fluorescence microscope. The length of the comet tail indicates the frequency of DNA breaks

The alkaline comet assay involves measurement of DNA damage in SSB and DSB. This method is fast and cheap. It provides important information about the risk of diseases related to oxidative stress (Alapetite *et al.*, 1999; Dusinska and Collins, 2008). In this assay, cells are embedded in a thin layer of agarose on a thin glass slide, cells are lysed in a solution containing detergent and NaCl, releasing the DNA from the proteins bound to it, but leaving DNA fragments still attached to the nuclear membrane. Then, the plate is incubated in an alkaline solution, an electrophoresis is run and DNA is stained with ethidium bromide. DNA fragments travel to the anode forming a comet-like image when viewed by fluorescence microscopy (Fikrová *et al.*, 2011, Baumgartner *et al.*, 2012). The image of the comet head denotes the DNA content and the tail the frequency of DNA breaks (Figure 2B). Software programs designed to analyze the comet image allow measurement of DNA content and tail length. The length of the comet tail correlates with the level of DNA damage.

Hair *et al.* (2010) used a modified comet assay method in which slides with cells embedded in agarose were incubated with three different treatments: 1) alkaline electrophoresis to detect SSB induced radiation and alkaline-labile sites; 2) electrophoresis of cells treated with formamidopyrimidine [Fapy] -DNA glycosylase (Fpg); this releases the damaged purines, leaving apurinic sites (AP sites) that are subsequently cleaved with the cellular AP lyase, producing single strand fragments which can be visualized in the comet assay, and 3) electrophoresis after treatment of the cells with bacterial endonuclease *Endo*III, which cleaves the damage strands at sites presenting oxidized pyrimidines, thus increasing the sensitivity of the comet assay by leaving gaps in mutated bases (Hair *et al.*, 2010).

Some disadvantages of the comet assay are the variability between different protocols and between laboratories, which makes it difficult to define ionizing radiation toxicities, so this issue will require adoption of standardized and comparable protocols (Forchhammer *et al.*, 2010; Henríquez-Hernández *et al.*, 2012; Azqueta *et al.*, 2014). Sirota *et al.* (2014) studied inter-laboratory variation of comet assay factors, like slide brands, duration of alkali treatment and electrophoresis conditions, and they found that laboratory differences were associated with electrophoresis conditions, especially the temperature during alkaline electrophoresis, which affects the rate of conversion of alkali labile sites to single stranded breaks (Sirota *et al.*, 2014). Additionally, it has been suggested that implementation of a standard software will be required for comet assay interpretation (Fikrová *et al.*, 2011).

### γ-H2AX

The histone H2AX variant of the histone H2A is present in subsets of nucleosomes (2 to 25% of the total H2A) and has been implicated in DSB repair. When H2AX is phosphorylated at the serine residue 139 by phosphoinositide-3-kinase-related protein kinases (PIKKs), the phosphate group adopts a γ position in the protein, constituting the gamma H2AX (γ-H2AX) configuration (Rogakou *et al.*, 1998; Rothkamm and Horn, 2009). This phosphoprotein acts in early events of DNA repair by decondensing the chromatin near the DSB (Kruhlak *et al.*, 2006). Additionally, γ H2AX joins to the DSB ends forming a “γH2AX focus” which is extended for several Mb at the sides of the DSB. A method used for the analysis of DNA damage is the measurement of γ-H2AX using antibodies against

In the γ-H2AX assays, peripheral blood is collected and mononuclear cells are separated and fixed on a glass surface. Then, an immunohistochemistry with anti-γ-H2AX antibody is performed and the results are analyzed by fluorescence microscopy in which fluorescent foci are measured (Figure 2A). This test may be also analyzed by flow cytometry or by western blot (Kinner *et al.*, 2008; Dickey *et al.*, 2009; Podhorecka *et al.*, 2010).

γ-H2AX foci measurements in patients before and after radiotherapies using low and high doses of ionizing radiation have shown a linear relationship between DNA damage and exposure to radiation. The initial number of γ-H2AX foci is consistent with DSBs in the cells. After a while, the γ-H2AX foci disappear due to the DNA repair (Rübe *et al.*, 2008; Horn *et al.*, 2011). This method is sensitive for measuring DNA repair in patients undergoing radiotherapy, but it is also applied in other fields, such as DNA damage analysis due to occupational exposure or contact with environmental pollutants, cigarette smoke, drugs, etc‥ It is important to note that these co-exposures may affect the results in radiotherapy patients and, hence, should be considered on an individual basis. Furthermore, phosphorylation of H2AX is observed in the absence of DSB in the replication process, in mitosis and during DNA fragmentation in apoptosis. Therefore, the test must be able to distinguish between apoptotic and non-apoptotic cells (Dickey *et al.*, 2009).

Comet assay and γ-H2AX methods described above help to assess DNA damage and repair, but do not allow discrimination of the type of damage, like SSB or DSB. It is also important to analyze whether the damage is repaired and what kind of repair mechanism is operating to assess whether cells are sensitive or resistant to ionizing radiation.

### Engineered proteins to detect spontaneous DSB

Shee *et al.* (2013) developed a new synthetic technology to quantify DSBs in bacterial and mammalian cells. This method use the green fluorescent-protein (GFP) fused to the GAM protein (GAM-GFP), a viral protein from bacteriophage Mu, which shares sequence homology with the eukaryotic proteins KU80 and KU70 involved in NHEJ (Aparicio *et al.*, 2014). Unlike the KU protein, the GAM protein is not involved in DNA repair reactions. GAM binds to DNA and inhibits a variety of exonucleases involved in DNA repair (Abraham and Symonds, 1990; Fagagna *et al.*, 2003; Shee *et al.*, 2013). This advance allows the study and quantification of DNA breaks. In this method, the I-*Sce*I endonuclease is used to make site specific DSBs and cells are transfected with a Mu GAM-GFP fusion expression vector. The GAM-GFP protein joins the DSBs formed by the I-*Sce*I treatment, generating fluorescence at the damaged sites which can be analyzed by fluorescence microscopy. Since the GAM-GFP protein competes with KU proteins, this results in low levels of DNA damage, thus limiting this technology to the study of DSB repair by HR (Shee *et al.*, 2013).

### Identification of repair mechanisms by specific DNA substrates

**Figure 3:**
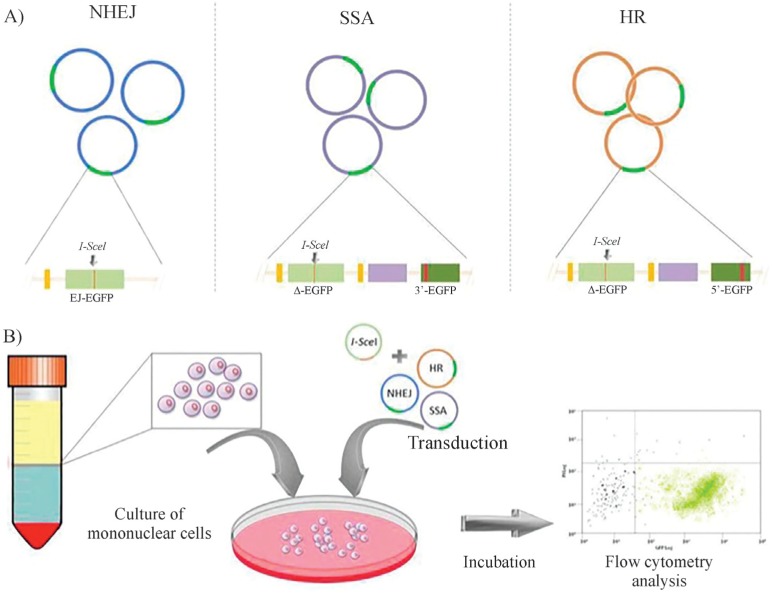
Specific assays for detecting DNA damage **(A)** The EJ-EGFP plasmids contains a mutated version of the EGFP gene (green light bar) created by inserting a restriction site for the meganuclease I-*Sce*I flanked by a 5 bp microhomology sites (black arrows); this plasmid was designed to be repaired by NHEJ. The Δ-EGFP/3’EGFP and Δ-EGFP/5’EGFP plasmids contain an array of an EGFP mutated gene containing an I-*Sce*I site (green light bar) followed by a spacer (purple bar) and EGFP gene versions truncated at their flanking 3’ and 5’ ends, respectively (dark green bars) which allow the reconstitution of the *wild-type* version of the marker gene by SSA and HR, respectively. **(B)** Analysis of DSB repair: The assay is performed in three cultures of peripheral blood lymphocytes (PBLs), transduced separately with each of the plasmid versions designed for discrimination of SSA, NHEJ and HR. The cultures are co-transduced with an additional plasmid expressing the I-*Sce*I enzyme. After generating DBS in the target plasmids by the expressed restriction enzyme, DNA repair in PBLs repair by each of the different DNA repair pathway may be monitored by restoration of the wild-type version of EGFP 24 h after transduction by measuring EGFP florescence by flow cytometry.

As mentioned above, Keimling *et al.* (2012) developed an *in vitro* method in which PBLs are transfected with marker plasmids for enabling discrimination of the mechanisms involved in DSB repair: HR, NHEJ, and SSA (Figure 3A). In this procedure, PBLs are transduced in three different experiments with separate plasmids, each containing the EGFP reporter gene followedby different sequences amenable to undergo one of the different mechanisms of DNA repair defined above. Cells in the three groups are co-transduced with a plasmid codifying for I-*Sce*I as the inductor of DSB repair events. Fluorescence detection after 24 h by flow cytometry in any of the three transduced cells of the panel measures the events of each individual operating mechanism, allowing more detailed information about DSB repair in individual patients (Figure 3B). This test is amenable for high-throughput sample processing and analysis (Boehden *et al.*, 2002; Keimling *et al.*, 2012).

## Conclusions

Detection of genetic alterations in genes associated with breast cancer, particularly genes related to DSB repair, may allow the diagnosis for genetic patients with breast cancer, but current methods based on genomic methodologies to detect mutations are expensive and not suitable for screening subjects under risk for increased DSB events. Almost 20% of the breast cancer patients will show acute complications due to radiotherapy. Hence, evaluation of DSB repair is a useful tool for assessing breast cancer risk and predicting the response and complications associated with conventional radiotherapy. Methods for studying DSB repair in PBLs are less expensive and suitable for designing high-throughput analyses for screening subjects at high risk for cancer in general, to anticipate adverse events and to offer individualized therapies. These methods will be relevant for preventing unnecessary radiation exposure, for screening of patients which will not benefit from radiotherapy, and for adjusting radiotherapy regimes in patients requiring this therapeutic option, in order to avoid adverse effects associated with DSB in tissues that can ameliorate a patient's prognosis.

A general comparison of methods shows that the comet assay assesses the amount of DNA damage, is inexpensive and is easy to perform in conventional laboratories. However it does not provide detailed information about the DNA lesion (SSB or DSB) and neither the DSB repair mechanism (NHEJ, SSA or HR). Another disadvantage of this method is the inter-protocol and the inter-laboratory variability in results. Nonetheless, this test is useful as a preliminary tool for assessing DNA damage. Detection of γ-H2AX is also a simple procedure and measurement of γ-H2AX may be performed by fluorescent microscopy, but the technique is also amenable for flow cytometry or western blot assays, which may render a more precise quantification than the comet assay. However, the detection of γ-H2AX does not discriminate between SSB and DSB. Furthermore, γ-H2AX may be phosphorylated during mitosis or apoptosis, resulting in false positives. The method developed by Shee *et al.* (2013) is more sensitive for DSB detection. It uses the GAM protein linked to EGFP, which joins the ends of the DSB and prevents DNA repair. Cells with DSB may be measured by fluorescent microscopy or flow cytometry. This technique requires molecular and cell biology techniques which may constitute an obstacle for diagnostic laboratories. The method developed by Keimling *et al.* (2012) enables the discrimination and measurement of the type of DSB repair mechanism. This method also uses techniques of molecular and cell biology, which may complicate its implementation in diagnostic laboratories, but this refined technology may have a great impact in defining a patient's risk to DSB induced by ionizing radiation.

Further advances in the discovery of genes involved in DNA repair and additional factors affecting genome stability will prompt the implementation of better technologies to study DNA damage in the clinical setting so as to avoid radiation-related toxicities.
